# “I Am Not Struggling Alone”: communicating support through peer mutual aid to reduce academic stress transmission and emotional contagion in large university classes

**DOI:** 10.3389/fpsyg.2026.1904847

**Published:** 2026-08-24

**Authors:** Shi Yin, Ruqiao Wu, Wenqi Ma, Yifan Ou

**Affiliations:** 1 School of Law, Chongqing University, Chongqing, China; 2 Faculty of Education, Northeast Normal University, Changchun, Jilin, China; 3 School of Social Sciences, University of Manchester, Manchester, United Kingdom; 4 Department of Communication Science, University of Antwerp, Antwerpen, Belgium

**Keywords:** academic stress transmission, emotional contagion, large university classes, peer mutual-aid, social interpretation, supportive communication

## Abstract

Large university classes are important communication environments in which students observe, interpret, and exchange stress-related information. Academic stress may therefore spread not only through individual experience but also through interpersonal communication and social interpretation,and shared meaning-making processes. How students communicate about academic challenges, interpret peers’ stress-related cues, and construct shared understandings of academic pressure may therefore influence whether stress becomes socially amplified or collectively managed. This study examined whether a brief peer mutual-aid routine was associated with lower academic stress transmission and negative emotional contagion by making academic struggle more visible, shareable, and collectively manageable. A two-wave quasi-experimental design was used with 460 students at T1 and 382 usable follow-up cases at T2. The intervention embedded small mutual-aid groups into large lecture sections through guided check-ins, reciprocal help-seeking norms, shared planning, and brief collective coping routines. Multi-item measures assessed peer support, academic stress, stress transmission, negative emotional contagion, class belonging, collective coping efficacy, help-seeking openness, and academic loneliness. Baseline-adjusted ANCOVA models indicated that students in peer mutual-aid sections reported higher T2 peer support, belonging, collective coping efficacy, and help-seeking openness, and lower T2 academic stress, stress transmission, negative emotional contagion, and academic loneliness than students in standard large lecture sections. Effects remained significant after multiple-comparison correction. The findings suggest that peer mutual aid in large classes may be associated with less spread of academic distress not by denying stress, but by reshaping the communication processes through which students encounter, interpret, negotiate, and collectively manage academic challenges. More broadly, the findings support the view that academic stress transmission is not merely a psychological phenomenon but also a communicative process embedded in everyday peer interaction.

## 1 Introduction

Large university courses are efficient organizational solutions, but they can also become socially and emotionally thin learning environments. A student may sit among hundreds of classmates and still experience academic pressure as a private failure, particularly when public signals of stress are more visible than public signals of help. This paradox has long been noted in higher education research: large classes can expand access and instructional efficiency while weakening student instructor contact, peer familiarity, and the informal relational cues through which students decide whether they belong and whether their struggle is normal (Tinto, 1997; Mulryan-Kyne, 2010). More recent work has similarly emphasized that student success depends not only on formal instruction, but also on the quality of relationships, recognition, and everyday connection within learning environments (Felten and Lambert, 2020; Ahn and Davis, 2023). In such settings, academic stress is not only an individual response to workload. It is also socially interpreted. Students observe peers rushing before deadlines, compare perceived progress, read anxious messages in course chats, and infer whether they are falling behind. These cues can make stress feel contagious even when the academic task itself has not changed. Social support theory suggests that support buffers stress by changing appraisal and perceived coping resources, while social learning theory explains how people acquire expectations, norms, and coping scripts through observing others (Cohen and Wills, 1985; Bandura, 1977). Peer motivation and peer learning perspectives extend this logic to educational settings, showing that students’ adjustment is shaped by the relational and normative cues they receive from classmates (Wentzel, 1998; Topping, 2005). Recent higher education research has further shown that belonging helps students interpret difficulty as a normal part of academic participation rather than as evidence that they do not fit (Allen et al., 2018; Gopalan and Brady, 2020). This is important because belonging is not merely a pleasant emotional state, but a social interpretation of whether one is recognized as a legitimate participant in the academic community (Pedler et al., 2022). Relationship rich education and peer connection research likewise suggest that repeated, meaningful contact with others can support persistence and engagement, especially when students might otherwise experience university learning as anonymous or fragmented (Caligiuri et al., 2020; Rasco et al., 2023). Intervention studies have also shown that relatively brief changes to students’ social context can influence motivation and equity related outcomes when they provide credible cues that struggle is common and support is available (Murphy et al., 2020; Binning et al., 2020). Yet the emotional side of large class learning remains undertheorized. Students do not experience academic stress in isolation. They continually encounter stress related information through classroom conversations, peer discussions, group chats, and observations of classmates’ emotional reactions. From a communication perspective, stress becomes meaningful through interpersonal interaction and social interpretation. Much of the intervention literature asks whether students feel more supported, belong more strongly, or perform better, but less often asks how students interpret the stress they see in others. This distinction matters because a large course can be academically demanding and still socially protective, or it can be academically demanding and socially amplifying. Emotional contagion research indicates that affective states can spread through mimicry, shared attention, and group interpretation (Hatfield et al., 1994; Barsade, 2002). Individual differences in susceptibility to emotional contagion also suggest that students may vary in how strongly they absorb or respond to others’ visible anxiety (Doherty, 1997). More recent work further shows that emotional contagion is not only automatic mimicry, but also depends on social attention, interpretation, and shared meaning within groups (Prochazkova and Kret, 2017). In classroom settings, visible anxiety may become personally threatening when students lack countervailing signals of support, normality, and collective coping. The central problem is therefore not simply whether students have stress, but whether stress is interpreted as isolated inadequacy or as a manageable academic condition shared with others.

The phrase “I am not struggling alone” captures the theoretical pivot of this study. Peer mutual-aid may matter not because it removes academic pressure, but because it changes the social meaning of pressure. When students can briefly name obstacles, exchange strategies, and see help-seeking modeled as ordinary, the same stress cue may be interpreted less as evidence of personal failure and more as part of a shared learning process. This interpretation aligns with classroom belonging and student retention research, where being recognized as a legitimate member of the learning community is associated with stronger engagement, adjustment, and persistence (Goodenow, 1993; Strayhorn, 2018). Recent studies similarly show that students’ sense of belonging is shaped by everyday relational experiences and can influence how they navigate academic difficulty, transition, and persistence (Meehan and Howells, 2019; Korpershoek et al., 2020). It also aligns with cooperative learning and collaborative learning scholarship, which argues that structured interdependence, not merely physical proximity, is what turns peer presence into a learning resource (Johnson et al., 1998; O’Donnell and Hmelo-Silver, 2013). Experimental and applied work on collaborative learning further suggests that interaction can improve learning when students jointly process information, compare understanding, and coordinate strategies rather than simply sit near one another (Tullis and Goldstone, 2020; Buchs et al., 2017). This matters especially for large lecture courses because the presence of many peers can paradoxically increase anonymity. Students may see hundreds of classmates but have no ordinary, low risk channel for asking whether others are also confused, delayed, or anxious. Without such channels, peer cues may become distorted: a classmate’s visible urgency can look like evidence that one is personally behind, and silence can look like evidence that everyone else is coping better. Relationship rich education, active learning research, and recent peer support studies suggest that even modest structured interaction can change these interpretations by making peer contact academically purposeful and socially legitimate (Felten and Lambert, 2020; Theobald et al., 2020). Evidence from online and blended learning during disrupted educational conditions also indicates that students’ social connection and perceived support are important for maintaining engagement when learning feels fragmented or isolating (Strelan et al., 2020; Mishra, 2020). Recent peer support research in higher education further suggests that structured peer contact can support adjustment, wellbeing, and help-seeking, particularly when students need low threshold forms of social and academic support (Pointon-Haas et al., 2023). However, peer support, belonging, and emotional contagion are often discussed in separate literatures. Peer support research has shown that social connection is associated with academic adjustment, mental wellbeing, and persistence, but it often treats support as a general individual resource rather than as a classroom level norm that can be deliberately structured (Mishra, 2020; Caligiuri et al., 2020). Other work likewise shows that peer support may protect students’ wellbeing, but it less often explains how support changes the interpretation of classmates’ stress cues inside specific course routines (Worsley et al., 2022; Pointon-Haas et al., 2023). Belonging research has demonstrated that students’ sense of being accepted and valued in the academic community predicts motivation and retention, but belonging interventions often focus on belief change rather than on repeated peer interaction inside day to day courses (Walton and Cohen, 2011; Allen et al., 2021a). Newer belonging studies continue to show that belonging is shaped by institutional and relational contexts, yet the link between belonging routines and classroom emotional dynamics remains underdeveloped (Gopalan and Brady, 2020; Pedler et al., 2022). Recent work also calls attention to belonging as an ongoing educational condition rather than a one time psychological message, which makes large class peer routines especially relevant (Haddow and Brodie, 2024). Emotional contagion research has explained how affect can move across people and groups, but it is less often connected to concrete course design routines that might redirect the social flow of worry, discouragement, and coping (Barsade, 2002; Goldenberg and Gross, 2020). Taken together with recent research on social attention and interpersonal affective transmission, this literature suggests that stress in large courses should be understood not only as an individual burden, but also as a communicative and relational process (Prochazkova and Kret, 2017). Bringing these strands together leads to a modest but educationally important proposition: if stress spreads through social interpretation, then peer routines that make support visible may alter stress transmission without pretending that demanding coursework has become easy.

**Figure 1:**
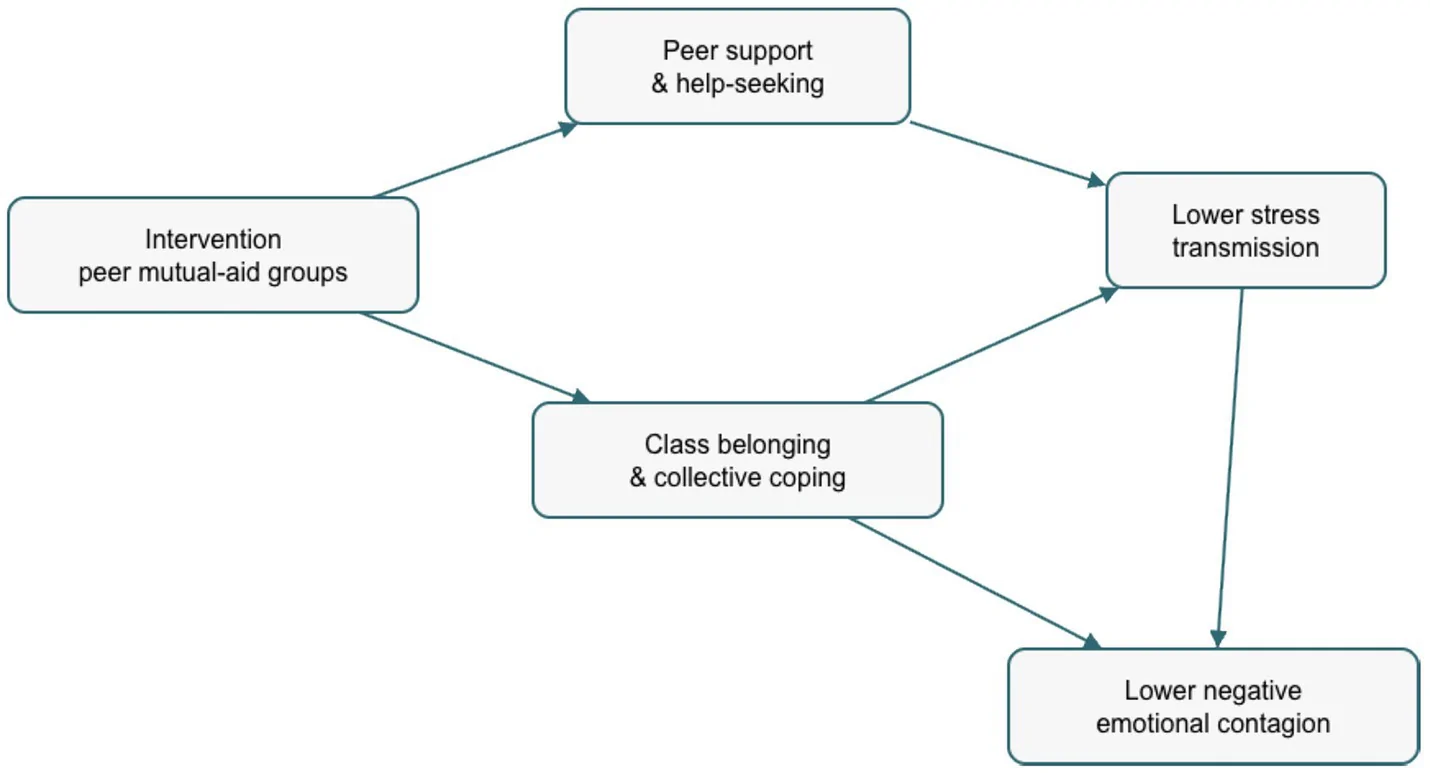
Conceptual model of peer mutual-aid, stress transmission, and emotional contagion.

The present study tests this proposition through a two-wave class-based quasi-experimental design in large university lecture sections. The central question is whether increasing structured peer contact amplifies shared stress or helps students interpret academic pressure as more manageable. The intervention created small mutual-aid units inside large classes over a four-week period. Students completed brief check-ins, named academic obstacles, exchanged concrete strategies, and practiced low-stakes help-seeking. The intervention was not counseling, clinical stress treatment, or a promise to eliminate stress. It was a classroom-climate routine designed to normalize shared coping and make ordinary academic difficulty more discussable before it escalated into silent distress. A low-intensity peer routine cannot replace professional advising, institutional support, or mental-health services, but it may provide a scalable relational structure in settings where individualized support is limited. The study included 460 students at baseline and 382 usable follow-up cases. Because assignment occurred by lecture section rather than individual randomization, the design is interpreted cautiously as a quasi-experimental classroom study (Figure 1).

The study contributes in three ways. First, it frames academic stress as a communicatively constructed social process rather than only an individual burden. Second, it tests peer mutual-aid as a practical classroom routine for large in-person lecture settings, where individualized support is often limited. Third, it distinguishes perceived peer support, stress transmission, negative emotional contagion, belonging, collective coping, help-seeking openness, and loneliness as related but separate classroom-climate outcomes. The contribution is therefore focused on baseline-adjusted class-level changes rather than on strong causal or mediation claims.

## 2 Theory and hypotheses

### 2.1 Academic stress as a socially transmitted experience

According to Social Information Processing Theory, individuals rely on social cues communicated by others to understand situations, construct meaning, and guide behavioral responses (Walther, 1992). In educational environments, students frequently observe and interpret information communicated by peers regarding workload, performance, deadlines, and coping strategies. These communicative cues may shape how academic stress is understood and experienced. From a communication perspective, stress-related cues do not carry fixed meanings. Rather, their significance is shaped through interpretation, discussion, and ongoing interaction with others. The same academic difficulty may therefore be experienced differently depending on whether students encounter communicative environments characterized by support, shared coping, and open help-seeking, or by silence, uncertainty, and negative comparison.

Academic stress is commonly measured as an individual state, but students often learn how stressed they should feel by observing others. Social-learning theory suggests that people infer norms, expectations, and likely consequences from the behavior and affect of those around them (Bandura, 1977). In university courses, this observational process is amplified by social comparison, shared deadlines, group chats, and visible study routines. Students may hear that everyone is behind, see peers studying late, or read anxious messages before deadlines. These cues can intensify pressure even when the student’s own workload has not changed. Stress-buffering theory helps explain why the same cue may have different consequences depending on whether support is available: stress becomes more threatening when students perceive fewer coping resources (Cohen and Wills, 1985). In large classes, the support side of this equation can be weak because students are physically proximate but socially anonymous (Mulryan-Kyne, 2010; Felten and Lambert, 2020). Stress transmission therefore refers here to the classroom process through which perceived peer stress increases one’s own stress appraisal. This construct is related to, but narrower than, general academic stress. It focuses on the social movement of pressure from classmates’ visible struggle to one’s own sense of threat. This distinction matters because an intervention may not substantially reduce course demands but may still change whether peer stress becomes personally contagious (Mishra, 2020; Havik and Westergard, 2020; Worsley et al., 2022).

### 2.2 Emotional contagion and the problem of silent struggle

Emotional contagion occurs when affective states spread through observation, interaction, mimicry, shared attention, or common interpretation. Classic work describes contagion as the tendency to catch others’ emotions through expressive and perceptual channels (Hatfield et al., 1994), and group research shows that shared affect can influence cooperation, conflict, and collective performance (Barsade, 2002). Doherty’s Emotional Contagion Scale further established that individuals differ in their susceptibility to others’ emotional displays (Doherty, 1997), while later neurocognitive and digital-contagion work emphasizes that contagion also depends on context, attention, and social meaning (Prochazkova and Kret, 2017; Goldenberg and Gross, 2020). In classrooms, negative emotional contagion may emerge when worry, discouragement, or panic becomes socially amplified. The risk is not simply that students see stress; stress is often visible in demanding educational environments. The risk is that visible stress is not accompanied by visible coping. If everyone seems overwhelmed and nobody explains how they are managing, distress can become both normalized and threatening. Large lectures can intensify this pattern because many students are exposed to the same deadlines and performance pressures but have limited channels for supportive interpretation (Tinto, 1997; Mulryan-Kyne, 2010). The present study therefore treats emotional contagion as a classroom-climate outcome. It asks whether a low-intensity peer routine is associated with lower negative emotional contagion by changing the social information students receive about struggle, support, and coping.

### 2.3 Peer mutual aid as a classroom intervention

Peer mutual-aid differs from general friendliness or unstructured peer contact. It involves structured reciprocity: students are prompted to check-in, share obstacles, exchange strategies, and make help-seeking normal. From a communication perspective, these activities create repeated opportunities for students to exchange stress-related information, disclose academic difficulties, and negotiate shared understandings of academic challenges. Through such communicative interactions, peer mutual-aid may reshape how stress cues are interpreted within the classroom environment. Cooperative-learning scholarship has long argued that positive interdependence and promotive interaction are more consequential than simple group placement (Johnson et al., 1998). Collaborative-learning research similarly shows that peer explanation, shared regulation, and mutual monitoring can support learning when interaction is structured around meaningful tasks (O’Donnell and Hmelo-Silver, 2013; Tullis and Goldstone, 2020). In a large lecture, this structure matters because students cannot rely on spontaneous intimacy or frequent instructor mediation. Mutual-aid routines make support predictable, low-cost, and legitimate. They also change the interpretation of stress: a difficult week becomes a shared academic condition rather than a private inadequacy. Recent higher-education work on relationship-rich education and peer support similarly argues that student success is shaped by repeated relational encounters, not only by formal academic services (Felten and Lambert, 2020; Mishra, 2020; Pointon-Haas et al., 2023). The intervention tested here was intentionally modest: brief weekly check-ins, obstacle naming, strategy exchange, and reciprocal help prompts. Its theoretical target was classroom climate rather than therapy. If students see that peers are struggling and coping, stress cues may become less isolating. Peer mutual-aid is therefore expected to predict higher perceived peer support and lower stress-related outcomes after baseline differences are controlled.

*H1:* Students in the peer mutual-aid intervention will report higher T2 peer support than students in the large lecture control condition.

*H2:* Students in the peer mutual-aid intervention will report lower T2 academic stress and stress transmission.

### 2.4 Belonging, collective coping, and help-seeking

Peer support may reduce distress because it increases students’ sense that they remain legitimate members of the class even when they struggle. This process is inherently communicative because feelings of belonging are often developed through repeated interaction, recognition, self-disclosure, and supportive exchanges with peers. Belonging has been consistently linked to motivation, engagement, retention, and well-being across educational contexts (Goodenow, 1993; Tinto, 1997; Strayhorn, 2018; Allen et al., 2018). In higher education, belonging is especially important during moments of uncertainty because students may interpret difficulty as evidence that they do not fit academically or socially (Walton and Cohen, 2011; Meehan and Howells, 2019; Gopalan and Brady, 2020). Recent belonging interventions show that changing the meaning of adversity can improve persistence and equity, particularly when students receive credible cues that struggle is common and temporary (Murphy et al., 2020; Binning et al., 2020). Peer mutual-aid offers a course-level route to this meaning change. It does not merely tell students that they belong; it gives them repeated small interactions in which belonging can be enacted. Collective coping efficacy is also relevant. Students may experience academic pressure differently when they believe classmates can exchange useful strategies and manage challenges together. Help-seeking openness completes the mechanism because students who see help as normal are less likely to hide problems until they escalate. However, belonging should be treated cautiously as a mechanism. It may improve as a result of peer routines, but its indirect effect on stress transmission must be evaluated empirically rather than assumed.

*H3:* Students in the peer mutual-aid intervention will report higher T2 class belonging, collective coping efficacy, and help-seeking openness.

### 2.5 Emotional contagion and loneliness

If peer mutual-aid changes the social meaning of stress, it should also be associated with lower negative emotional contagion and academic loneliness. The expected effect on emotional contagion is not that peers stop feeling stressed. Rather, stress cues should become less threatening when accompanied by support, explanation, and shared coping. This claim is consistent with emotional-contagion theory, which emphasizes that affect spreads through interpersonal perception, but also with classroom-belonging research, which suggests that social interpretation can moderate the meaning of difficulty (Hatfield et al., 1994; Barsade, 2002; Allen et al., 2021b; Korpershoek et al., 2020). Loneliness is included because large classes can produce a specific kind of academic isolation: students may be surrounded by people while lacking a reliable partner for interpreting obstacles. Prior work on peer relationships and university adjustment suggests that peer connection can reduce this sense of isolation and support adaptation to higher education (Maunder, 2018; Pedler et al., 2022; Felten and Lambert, 2020). A mutual-aid routine may reduce loneliness by making classmates’ availability visible and by establishing repeated contact around concrete academic tasks. Again, the prediction is modest. The intervention is not expected to remove all loneliness or distress, but students in peer mutual-aid sections should report lower negative emotional contagion and academic loneliness at follow-up when compared with control sections after baseline adjustment.

*H4:* Students in the peer mutual-aid intervention will report lower T2 negative emotional contagion and academic loneliness.

## 3 Method

### 3.1 Design and participants

**Figure 2:**
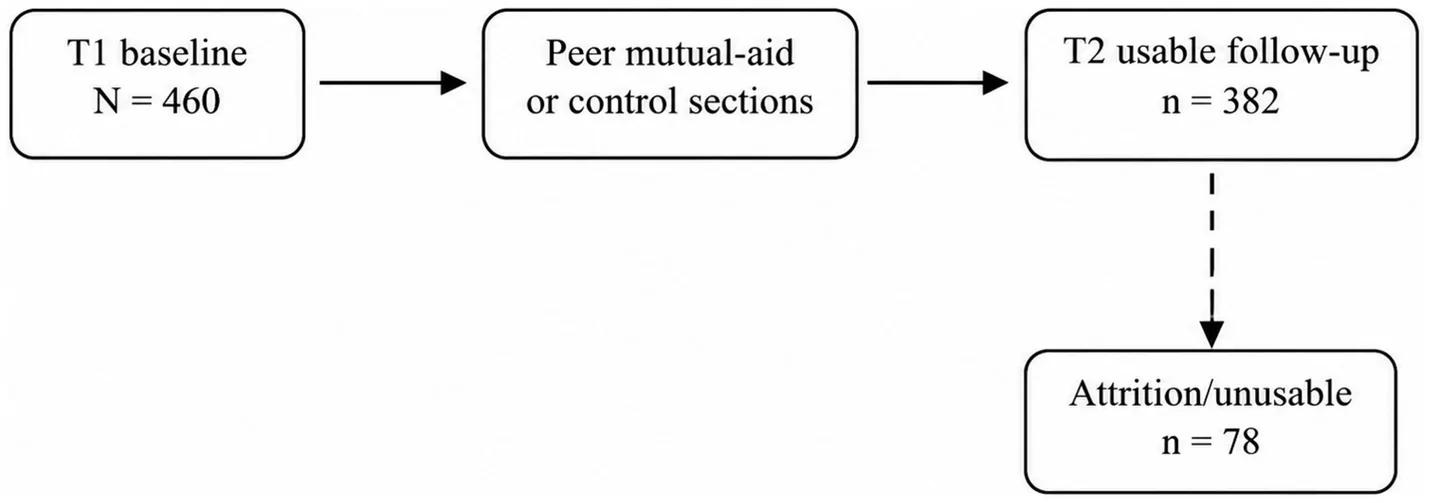
Two-wave sample flow.

The study used a two-wave class-based panel design with a four-week interval between T1 and T2. Participants were recruited from large undergraduate lecture sections at a university in southwestern China, including general education and social science related courses. The T1 baseline sample included 460 students. The intervention was implemented by lecture section: some sections received the peer mutual-aid routine, whereas comparable sections continued with standard large-class practice. The sections followed broadly similar course schedules, teaching formats, and assessment arrangements. Because assignment occurred at the lecture-section level rather than through individual randomization, the design was treated as quasi-experimental. Baseline balance checks and covariate-adjusted models were used to reduce possible selection differences between groups. All students provided informed consent before participation. Participation in the research surveys was voluntary and did not affect course grades. At T2, 382 students remained in the usable follow-up sample after withdrawal, missing follow-up data, and invalid response screening. The retained sample included 191 students in the control sections and 191 students in the peer mutual-aid sections. Figure 2 reports the sample flow.

### 3.2 Intervention

The peer mutual-aid intervention embedded small support units into large lecture sections. Students in the intervention sections were assigned to small groups and completed the same brief routine once per week across the four-week intervention period. Each weekly routine lasted approximately 10–15 min and followed a common sequence: a short check-in, naming one current academic obstacle, exchanging one concrete strategy, identifying one upcoming deadline, and making one help request or help offer. The routines were introduced by course instructors and completed during or immediately after regular class sessions. The intervention was not designed as counseling or clinical stress treatment. It was a low-intensity classroom routine intended to normalize help-seeking and shared coping within large lecture environments. It did not replace instruction, grading, advising, or formal counseling. Its purpose was to make academic struggle more visible and peer support more routine in a setting where students could otherwise remain socially anonymous.

Control sections received the same course content, assignments, and assessment schedule but continued standard large lecture practice. They did not complete structured peer mutual-aid routines or an equivalent active attention control activity. Intervention fidelity was tracked weekly using check-in completion, group activity ratings, strategy exchange counts, and a composite fidelity index. These indicators were used to describe implementation quality rather than to exclude students from the intervention condition. This design allowed the study to examine the peer mutual-aid routine under realistic classroom conditions, while also acknowledging that the estimated effects may partly reflect the additional structured interaction time provided to intervention sections.

### 3.3 Measures

All focal constructs used seven-point Likert-type items. Peer mutual-aid support was measured with five items about availability, reciprocity, encouragement, and practical help, drawing on the stress-buffering and peer-support traditions (Cohen and Wills, 1985; Topping, 2005; Mishra, 2020). Academic stress used five items about workload pressure, worry, and overload, adapted for the course context from established academic-stress measurement traditions (Kohn and Frazer, 1986). Stress transmission used four classroom-adapted items based on stress-buffering, group-affect, and classroom belonging literatures to capture the extent to which students felt classmates’ stress became personally pressuring (Cohen and Wills, 1985; Barsade, 2002; Goodenow, 1993). Negative emotional contagion used five items adapted from the emotional contagion tradition, especially Doherty’s Emotional Contagion Scale and later group-affect research, but reworded for academic classroom contexts (Doherty, 1997; Hatfield et al., 1994; Goldenberg and Gross, 2020). Class belonging, collective coping efficacy, help-seeking openness, and academic loneliness were measured with four items each, informed by classroom belonging, cooperative learning, and university adjustment research (Goodenow, 1993; Strayhorn, 2018; Johnson et al., 1998; Maunder, 2018). Items were reviewed for classroom relevance and readability before administration and pilot checked with a small group of students to ensure that the wording was understandable in large-course settings. Because several items were adapted to the classroom context, the Results report reliability, correlations among scale scores, a core two-factor structure check, and common-method diagnostics before interpreting the intervention models. Full item wording is provided in Appendix A.

### 3.4 Analytic strategy

Analyses began from item-level data. Scale scores were computed as item means. The workflow examined retention, baseline balance, reliability, pre-post change, baseline-adjusted ANCOVA models, moderation, robustness checks, zero-order correlations, common-method diagnostics, and multiplicity correction. ANCOVA models predicted T2 outcomes from intervention condition while controlling the corresponding T1 outcome, prior GPA, course load, and first-generation status. Because eight outcome models were tested, false-discovery-rate and Bonferroni adjusted *p* values were reported for the main ANCOVA table. Change-score mediation was not interpreted as confirmatory evidence because two-wave raw change-score mediation cannot establish temporal precedence and may be confounded with baseline levels. The figures and tables were generated from the same analytic files (Table 1).

**Table 1:** Sample retention.

Stage	*N*	Percent	Notes
T1 baseline	460	100.0%	Students enrolled in large lecture sections completed the baseline survey.
T2 usable follow-up	382	83.0%	Students retained after withdrawal, missing follow-up data, and invalid response screening.
Attrition/unusable T2	78	17.0%	Not included in longitudinal analyses.

## 4 Results

### 4.1 Sample retention and baseline balance

The baseline sample included 460 students enrolled in large lecture sections. At T2, 382 students provided usable follow-up data, yielding a retention rate of 83.0%. The remaining 78 cases were excluded from longitudinal analyses because of withdrawal, missing follow-up data, or invalid response screening. This level of retention is acceptable for a two-wave class-based intervention study, although the possibility of selective attrition is considered in the subsequent baseline-balance and limitation sections (Table 2).

**Table 2:** Baseline balance.

Variable	Control M(SD)	Intervention M(SD)	*t*	*p*	Cohen *d*
Peer mutual-aid support	4.00 (0.63)	4.00 (0.66)	−0.13	0.897	−0.012
Academic stress	4.65 (0.63)	4.56 (0.63)	−1.506	0.133	−0.14
Stress transmission	4.25 (0.72)	4.27 (0.72)	0.182	0.856	0.017
Negative emotional contagion	4.37 (0.67)	4.34 (0.70)	−0.404	0.686	−0.038
Class belonging	3.87 (0.67)	3.77 (0.69)	−1.529	0.127	−0.143
Collective coping efficacy	3.83 (0.65)	3.90 (0.61)	1.179	0.239	0.11
Prior GPA	3.14 (0.38)	3.16 (0.38)	0.399	0.69	0.037
Course load	4.68 (0.98)	4.77 (0.99)	0.941	0.347	0.088

*t* values reflect Intervention minus Control (i.e., positive values indicate higher scores in the intervention condition; negative values indicate higher scores in the control condition). Cohen’s *d* is signed in the same direction.

Baseline comparisons suggested that the control and intervention sections were broadly comparable before the intervention. None of the baseline differences reached statistical significance, and the standardized mean differences were small across peer support, academic stress, stress transmission, negative emotional contagion, class belonging, collective coping efficacy, prior GPA, and course load. These results reduce, but do not eliminate, concern about initial group imbalance. To further address possible baseline differences, all main ANCOVA models controlled the corresponding T1 outcome and key covariates.

### 4.2 Reliability and pre-post change

Table 3 reports descriptive statistics and internal consistency estimates for the main study variables. Cronbach’s alpha values ranged from 0.662 to 0.790 across waves, indicating acceptable internal consistency for the multi-item scales. Mean-level patterns were generally consistent with the expected direction of change: peer support, class belonging, and collective coping efficacy were higher at T2, whereas academic stress, stress transmission, negative emotional contagion, and academic loneliness were lower. These descriptive patterns provide preliminary support for the subsequent adjusted models, although inferential conclusions are based on the ANCOVA and mediation analyses reported below.

**Table 3:** Descriptive statistics and reliability.

Construct	Wave	*N*	M	SD	Cronbach alpha
Peer mutual-aid support	T1	460	4.0	0.65	0.763
Peer mutual-aid support	T2	382	4.13	0.63	0.766
Academic stress	T1	460	4.61	0.63	0.758
Academic stress	T2	382	4.18	0.59	0.716
Stress transmission	T1	460	4.26	0.71	0.719
Stress transmission	T2	382	3.99	0.67	0.718
Negative emotional contagion	T1	460	4.36	0.69	0.79
Negative emotional contagion	T2	382	4.07	0.64	0.749
Class belonging	T1	460	3.82	0.69	0.758
Class belonging	T2	382	4.08	0.64	0.699
Collective coping efficacy	T1	460	3.87	0.63	0.709
Collective coping efficacy	T2	382	4.07	0.64	0.724
Help-seeking openness	T1	460	4.07	0.67	0.744
Help-seeking openness	T2	382	4.1	0.64	0.662
Academic loneliness	T1	460	4.22	0.76	0.785
Academic loneliness	T2	382	3.96	0.66	0.752

Table 4 presents paired pre-post changes among students retained at both waves. The intervention group showed increases in peer mutual-aid support, class belonging, collective coping efficacy, and help-seeking openness, alongside decreases in academic stress, stress transmission, negative emotional contagion, and academic loneliness. In contrast, the control group showed smaller and less consistent changes, with little change in stress transmission, class belonging, help-seeking openness, or loneliness. These unadjusted paired comparisons provide descriptive evidence of differential change, but they should be interpreted cautiously because the main tests of intervention effects rely on baseline-adjusted ANCOVA models. T1 and T2 means in this table are based on the retained paired sample with usable data at both waves (*N* = 382; control = 191, intervention = 191), rather than the full T1 baseline sample.

**Table 4:** Pre-post change by condition (retained paired sample).

Outcome	Condition	*N*	T1 M	T2 M	Change	*t*	*p*
Peer mutual-aid support	Large lecture control	191	4.09	4.0	−0.09	−1.972	0.050
Peer mutual-aid support	Peer mutual-aid intervention	191	4.07	4.27	0.19	4.133	<0.001
Academic stress	Large lecture control	191	4.57	4.31	−0.25	−5.875	<0.001
Academic stress	Peer mutual-aid intervention	191	4.51	4.05	−0.46	−10.527	<0.001
Stress transmission	Large lecture control	191	4.19	4.17	−0.02	−0.473	0.637
Stress transmission	Peer mutual-aid intervention	191	4.23	3.82	−0.41	−8.509	<0.001
Negative emotional contagion	Large lecture control	191	4.3	4.17	−0.13	−2.889	0.004
Negative emotional contagion	Peer mutual-aid intervention	191	4.29	3.98	−0.31	−7.128	<0.001
Class belonging	Large lecture control	191	3.96	3.92	−0.04	−0.933	0.352
Class belonging	Peer mutual-aid intervention	191	3.84	4.24	0.41	8.698	<0.001
Collective coping efficacy	Large lecture control	191	3.86	3.96	0.1	2.056	0.041
Collective coping efficacy	Peer mutual-aid intervention	191	3.94	4.18	0.24	5.006	<0.001
Help-seeking openness	Large lecture control	191	4.08	4.01	−0.07	−1.409	0.161
Help-seeking openness	Peer mutual-aid intervention	191	4.06	4.18	0.13	2.569	0.011
Academic loneliness	Large lecture control	191	4.15	4.14	−0.0	−0.078	0.938
Academic loneliness	Peer mutual-aid intervention	191	4.11	3.79	−0.33	−6.162	<0.001

Change scores were computed from unrounded means; displayed means are rounded to two decimal places, so the Change column may differ from the displayed T2 M minus T1 M by ±0.01.

### 4.3 Intervention effects

**Figure 3:**
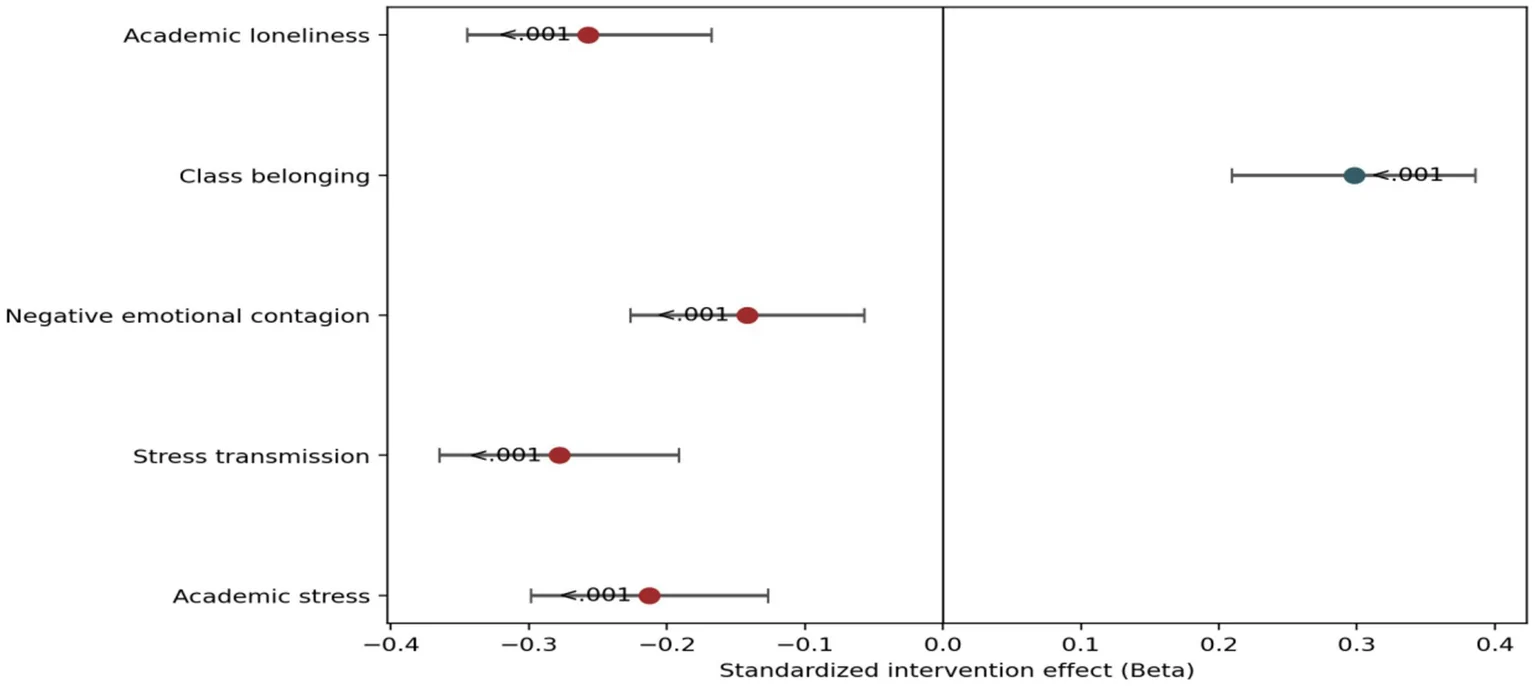
Adjusted intervention effects. Positive values indicate higher adjusted T2 outcomes in the peer mutual-aid intervention condition; negative values indicate lower adjusted T2 outcomes relative to large lecture control sections.

Figure 3 visualizes the standardized intervention effects and approximate 95% confidence intervals from Table 5 for the main stress and social–emotional outcomes. Positive values indicate higher adjusted T2 outcomes in the intervention condition, whereas negative values indicate lower adjusted T2 outcomes.

**Table 5:** ANCOVA models for T2 outcomes.

Outcome	Predictor	Beta	B	SE	95% CI	*p*	FDR *p*	Bonferroni *p*	Baseline controlled
Peer mutual-aid support	Peer mutual-aid intervention	0.212	0.268	0.057	[0.157, 0.379]	<0.001	<0.001	<0.001	Yes
Academic stress	Peer mutual-aid intervention	−0.213	−0.25	0.051	[−0.351, −0.149]	<0.001	<0.001	<0.001	Yes
Stress transmission	Peer mutual-aid intervention	−0.278	−0.374	0.059	[−0.491, −0.258]	<0.001	<0.001	<0.001	Yes
Negative emotional contagion	Peer mutual-aid intervention	−0.142	−0.181	0.055	[−0.289, −0.073]	<0.001	0.001	0.008	Yes
Class belonging	Peer mutual-aid intervention	0.298	0.383	0.058	[0.269, 0.496]	<0.001	<0.001	<0.001	Yes
Collective coping efficacy	Peer mutual-aid intervention	0.143	0.182	0.058	[0.069, 0.296]	0.002	0.002	0.013	Yes
Help-seeking openness	Peer mutual-aid intervention	0.149	0.189	0.06	[0.072, 0.306]	0.002	0.002	0.013	Yes
Academic loneliness	Peer mutual-aid intervention	−0.257	−0.341	0.06	[−0.458, −0.223]	<0.001	<0.001	<0.001	Yes

Table 5 reports the baseline-adjusted ANCOVA models for T2 outcomes. After controlling the corresponding T1 outcome and covariates, students in the peer mutual-aid condition reported higher peer support, class belonging, collective coping efficacy, and help-seeking openness at T2. They also reported lower academic stress, stress transmission, negative emotional contagion, and academic loneliness. The standardized effects were small to moderate in size, ranging from *β* = −0.142 for negative emotional contagion to *β* = 0.298 for class belonging. All eight intervention-condition coefficients remained statistically significant after FDR adjustment and Bonferroni correction, suggesting that the overall pattern was not driven by uncorrected multiple testing.

### 4.4 Correlation and common-method diagnostics

Table 6 summarizes key zero-order associations and a common-method diagnostic computed from the retained item-level data. The correlations were consistent with the expected classroom-climate pattern: peer support was negatively associated with stress transmission and academic stress, stress transmission was positively associated with negative emotional contagion, and class belonging was negatively associated with academic loneliness. Harman single-factor screening indicated that the first unrotated factor accounted for 11.3% of item variance, below the conventional 50% heuristic. This diagnostic reduces concern about a single dominant response factor, but it does not eliminate possible common-method bias because all focal outcomes were self-reported.

**Table 6:** Zero-order correlations and common-method diagnostics.

Evidence	*N*/items	Statistic	Value	Additional value	Interpretation
Peer support T2 with stress transmission T2	382	*r*	−0.284		Higher peer support was associated with lower stress transmission
Peer support T2 with academic stress T2	382	*r*	−0.197		Higher peer support was associated with lower academic stress
Stress transmission T2 with negative emotional contagion T2	382	*r*	0.314		Related but not redundant stress-related outcomes
Class belonging T2 with academic loneliness T2	382	*r*	−0.377		Higher belonging was associated with lower loneliness
Harman single-factor diagnostic	382/70 items	First eigenvalue	7.904	11.3% variance	No dominant single factor was observed
CMV interpretation	Self-report data	Diagnostic only	–	–	Common-method bias cannot be eliminated by this test

### 4.5 Moderation and robustness

Table 7 presents exploratory moderation tests for baseline academic stress. None of the interaction terms reached statistical significance for stress transmission, negative emotional contagion, or academic loneliness. The data therefore did not indicate that initially higher-stress students showed stronger intervention associations. A cautious interpretation is that the routine may have functioned as a broad classroom-climate practice rather than as a targeted support mechanism only for initially high-stress students.

**Table 7:** Moderation tests.

Outcome	Predictor	Beta	B	SE	95% CI	*p*
Stress transmission	Intervention × baseline academic stress	−0.005	−0.008	0.095	[−0.194, 0.178]	0.934
Negative emotional contagion	Intervention × baseline academic stress	−0.043	−0.061	0.096	[−0.249, 0.127]	0.523
Academic loneliness	Intervention × baseline academic stress	−0.033	−0.049	0.095	[−0.235, 0.137]	0.605

Table 8 reports robustness checks for the main stress-transmission result and the emotional-contagion outcome. The intervention coefficient for stress transmission remained negative and statistically significant across the unadjusted T2 comparison, baseline-only adjustment, the main covariate-adjusted model, additional adjustment for baseline academic stress, trimming of extreme stress-change cases, and change-score modeling. The emotional contagion model also showed a negative and statistically significant coefficient, although its magnitude was smaller. Across these specifications, negative coefficients indicate lower stress-related outcomes in the peer mutual-aid intervention condition relative to the control condition.

**Table 8:** Robustness checks.

Specification	*N*	B	SE	95% CI	*p*
Unadjusted T2 comparison	382	−0.348	0.067	[−0.479, −0.217]	<0.001
Baseline-only adjustment	382	−0.364	0.059	[−0.481, −0.248]	<0.001
Main stress transmission model	382	−0.374	0.059	[−0.491, −0.258]	<0.001
Add academic-stress baseline	382	−0.372	0.059	[−0.488, −0.255]	<0.001
Trim extreme stress-change cases	367	−0.343	0.056	[−0.453, −0.234]	<0.001
Change-score model	382	−0.389	0.072	[−0.530, −0.247]	<0.001
Main emotional contagion model	382	−0.181	0.055	[−0.289, −0.073]	<0.001

### 4.6 Fidelity and supplemental diagnostics

Table 9 summarizes implementation fidelity within the intervention condition. Fidelity was adequate but uneven, which is consistent with a low-intensity classroom-based intervention delivered in ordinary lecture settings. Students completed approximately 80% of the expected weekly check-ins on average, reported moderately high group activity, and completed about three of the four planned strategy exchanges. The observed variation in completion and activity ratings suggests that some mutual-aid groups engaged more fully than others, a point considered in the discussion when interpreting the size and stability of the intervention effects. These fidelity indicators were used as implementation descriptors only. The available item-level analytic file did not contain individually linked fidelity-dose variables, so the manuscript does not estimate fidelity-outcome associations or treat fidelity as a predictor of student outcomes. This limitation is addressed in the Discussion.

**Table 9:** Intervention fidelity.

Fidelity indicator	*N*	M	SD	Minimum	Maximum
Weekly check-in completion proportion	191	0.8	0.13	0.43	1.0
Group activity rating (1–7)	191	5.48	0.82	2.79	7.0
Strategy exchanges completed (0–4)	191	3.06	0.71	1.0	4.0
Composite fidelity index (1–7)	191	5.48	0.54	4.19	6.7

Supplemental diagnostics supported the main interpretation without expanding the table count. The core structure check for stress transmission and negative emotional contagion showed acceptable, though not uniformly strong, fit at both waves, supporting their treatment as related but distinguishable classroom-adapted constructs. Multiple-comparison checks showed that all eight ANCOVA intervention effects remained statistically significant after FDR correction and Bonferroni correction; therefore, the main intervention pattern was not solely a product of unadjusted multiple testing.

## 5 Discussion

### 5.1 Main findings

The findings suggest that modest peer mutual-aid routines may shape the social environment of large lecture classes in psychologically meaningful ways. Students in the intervention condition reported higher peer support, stronger belonging, greater confidence in handling academic pressure together, and greater openness to seeking help, alongside lower academic stress, stress transmission, negative emotional contagion, and academic loneliness after baseline adjustment. The standardized effects were generally small to moderate, which is consistent with the low-intensity and classroom-based nature of the intervention.

The results should not be interpreted as evidence that peer mutual-aid removed academic pressure itself. Coursework demands, deadlines, and evaluative stress remained largely unchanged across sections. Instead, the findings are more consistent with a shift in how stress was socially interpreted within the learning environment. This interpretation extends stress-buffering theory by showing that support may matter not only as practical assistance but also as a communicative cue that changes how students appraise shared difficulty (Cohen and Wills, 1985). It also qualifies emotional-contagion theory: peer contact does not necessarily amplify distress when interaction is structured around coping, reciprocity, and normalization. Thus, the study engages the literature critically by distinguishing beneficial structured peer contact from unstructured exposure to classmates’ stress cues.

### 5.2 Classroom implementation and practical implications

The implementation patterns observed in the present study suggest that the intervention functioned as a realistic educational routine rather than as a tightly controlled experimental program. Participation levels were adequate overall, but engagement varied across groups, and not all students completed every expected interaction or weekly check-in activity. This uneven participation is important because it reflects the practical conditions under which large university courses actually operate.

The routine should be understood as a complement to institutional support, not as a substitute for advising, counseling, disability services, instructor feedback, or structural improvements in course design. Large lecture courses often make stress highly visible while leaving support comparatively invisible. Under these conditions, brief opportunities for structured interaction may alter how students interpret ordinary academic pressure, but they cannot reliably improve learning outcomes by themselves. Future research could also examine adapted versions in asynchronous online courses, where peer cues, silence, and help-seeking norms may operate through discussion boards, group chats, and learning-platform interactions rather than face-to-face routines.

### 5.3 Limitations and future research

Several limitations should be acknowledged. First, the study used a quasi-experimental design based on existing lecture sections rather than individual random assignment. Although baseline balance checks and covariate-adjusted models reduced some concerns about initial group differences, unobserved section-level factors may still have influenced the findings. Instructor communication style, assessment timing, course norms, and prior peer familiarity could all shape how students experienced the intervention and how they interpreted classmates’ stress.

Second, the control sections continued standard large lecture practice and did not receive an equivalent active comparison activity. As a result, the observed effects may reflect both the specific peer mutual-aid mechanism and the additional structured interaction time received by intervention students. Third, the follow-up period was short. A four-week design is suitable for detecting short-term changes in peer support and stress interpretation, but it cannot show whether these changes persist across a semester or translate into academic performance, retention, or longer-term wellbeing. Fourth, the study relied on self-report measures. Although reliability, correlations, structure checks, and CMV diagnostics were reported, future research should add peer nominations, learning-platform data, classroom observation, and social-network indicators. Fifth, the sample came from one university in southwestern China. Norms surrounding help-seeking, peer obligation, modesty, and collective coping may differ across cultural and educational contexts, so generalization to other institutions, countries, or online programs requires caution. Sixth, implementation fidelity was described but not modeled as an outcome predictor because individually linked fidelity-dose variables were not available in the analytic file. Future studies should test whether completion rates, group activity quality, and strategy exchanges are associated with stronger student outcomes.

## 6 Conclusion

Large classes do not have to leave students socially isolated in their academic stress. This study suggests that structured peer mutual-aid was associated with lower stress transmission and negative emotional contagion by making support more visible, reciprocal, and routine within large lecture settings. The practical implication is modest: brief peer-support routines may help some students interpret academic pressure as a shared and manageable learning condition, but they should complement rather than replace institutional support, professional advising, and course-level improvements. Stress may still remain, and stronger designs are needed before claims can be made about durable learning outcomes.

More broadly, this study highlights the role of communication in shaping how academic stress is interpreted and shared within large classrooms. When opportunities for peer interaction, help-seeking, and strategy exchange are made visible, academic pressure may be experienced as a shared challenge rather than a private inadequacy.

## Data Availability

The raw data supporting the conclusions of this article will be made available by the authors, without undue reservation.

## Appendix A. Full item wording

All items used a seven-point response scale from 1 = strongly disagree to 7 = strongly agree.

Peer mutual-aid support: classmates in this course were available when I needed help; classmates encouraged one another; classmates exchanged useful strategies; I could ask peers for practical support; peer support in this class felt reciprocal.

Academic stress: course demands felt stressful; deadlines created pressure; I worried about keeping up; academic tasks felt overwhelming; I felt burdened by coursework.

Stress transmission: classmates’ stress made me feel more pressured; seeing peers struggle increased my own stress; anxious peer messages made academic pressure feel stronger; classmates’ stress made course demands feel harder to manage.

Negative emotional contagion: classmates’ worry affected my mood; I became tense when peers seemed tense; discouragement in the class spread to me; others’ anxiety made me anxious; negative emotions in the class were easy to absorb.

Class belonging: I felt like a legitimate member of the class; I felt accepted by classmates; I felt connected to the class; I felt that I belonged in the learning community.

Collective coping efficacy: classmates and I could handle academic challenges together; our group could exchange useful solutions; we could manage deadlines through shared planning; peer strategies helped us cope with course demands.

Help-seeking openness: asking classmates for help felt acceptable; I felt comfortable telling peers when I was stuck; help-seeking was normal in this class; I was willing to request academic support from classmates.

Academic loneliness: I felt alone with academic difficulty; I lacked classmates to talk with about course problems; I felt isolated in handling coursework; I had few peer connections for academic support.
